# Antiviral activity of mink interferon alpha expressed in the yeast *Pichia pastoris*

**DOI:** 10.3389/fvets.2022.976347

**Published:** 2022-09-14

**Authors:** Hailing Zhang, Dongliang Zhang, Han Lu, Deying Zou, Bo Hu, Shizhen Lian, Shiying Lu

**Affiliations:** ^1^Key Laboratory of Zoonosis, Ministry of Education, College of Animal Science and Veterinary Medicine, Institute of Zoonosis, Jilin University, Changchun, China; ^2^Key Laboratory of Special Animal Epidemic Disease, Ministry of Agriculture, Institute of Special Animal and Plant Sciences, Chinese Academy of Agricultural Sciences, Changchun, China

**Keywords:** mink, interferon-alpha, yeast expression, antiviral activity, serum drug concentration

## Abstract

Many viruses can cause infections in mink, including canine distemper virus, mink enteritis virus, and Aleutian disease virus. Current treatments are ineffective, and these infections are often fatal, causing severe economic losses. As antiviral drugs may effectively prevent and control these infections, recent research has increasingly focused on antiviral interferons. Herein, the gene encoding a mature mink interferon alpha (MiIFN-α) was synthesized according to the *P. pastoris* preference of codon usage and a recombinant plasmid, pPICZαA-MiIFN-α, was constructed. pPICZαA-MiIFN-α was linearized and transformed into the *P. pastoris* X33 strain, and zeocin-resistant transformants were selected. Protein expression was induced by methanol. SDS-PAGE and western blot analyses showed that a 25-kDa fusion protein was expressed in the culture supernatant. Antiviral activity of the expressed protein was determined using cytopathic effect inhibition (CPEI). The purified MiIFN-α significantly inhibited the cytopathic effect of vesicular stomatitis virus with a green fluorescent protein (VSV-GFP) in F81 feline kidney cells, with an antiviral activity of 6.4 × 10^7^ IU/mL; it also significantly inhibited MEV replication in F81 cells. MiIFN-α antiviral activity against VSV-GFP was significantly reduced on treatment with pH 4 and pH 10 conditions for 24 h (*p* < 0.01). Serum MiIFN-α concentrations in rat were measured using enzyme-linked immune-sorbent assay; MiIFN-α concentrations in rat serum peaked at ~36 h after injection. A high dose of MiIFN-α was safe for use. There were no significant differences in body temperature, tissue changes, and lymphocyte, total white blood cell, and central granulocyte counts between the injected and control groups (*p* > 0.05). These findings lay a foundation for the large-scale production of recombinant MiIFNs.

## Introduction

Although the prevalence of animal breeding for fur has declined over recent years, China remains one of the few countries with a developed fur animal breeding industry, with a stock of 8 million minks. The mink breeding industry is an important component of the rural industrial structure in China. However, frequent infectious outbreaks of canine distemper, mink viral enteritis, and Aleutian virus disease in the mink industry have resulted in serious economic losses. These viral infectious diseases are characterized by acute onset, high mortality, and unsatisfactory therapeutic effect of common drugs.

Interferons (IFNs) are the first line of defense against viral infections (1). Toll-like receptor (TLR)-3 and 4, RIG-I-like receptor, and the intracellular DNA receptor cyclic GMP-AMP synthase (cGAS) induce the expression of type I IFNs by activating TBK1 and IRF3. Upon pathogen infection, TLRs identify pathogen-associated molecular patterns, activate downstream signaling pathways, and induce the expression of pro-inflammatory factors and IFNs (2, 3). IFNs bind to IFN-α receptors and stimulate antiviral immunity by upregulating IFN-stimulated genes (4–8).

Research on animal IFNs has made some progress, but mainly focuses on a few animals. Several IFNs have been expressed and characterized to date (9); at present, recombinant IFN products for animals such as cats, dogs, and chickens are available commercially. However, few studies have investigated the characteristics of mink IFNs (MiIFNs). Mink IFN-α is a protein family encoded by 13 functional genes, which share similar lengths and amino acid sequence homology of 88.8–98.4%. The mature MiIFN-α contains 167 amino acids, and the signal peptide comprises 23 amino acids (10). Nonetheless, as yet, there has been no domestic production of genetically engineered mink IFN antiviral agents or the joint use of related products.

Given the large number and considerable severity of viral diseases in mink, it is essential to develop genetically engineered IFNs with broad-spectrum antiviral activity. However, traditional expression systems are inefficient and complex; thus, they are difficult to use at a large scale. Therefore, identifying efficient methods for IFN production is critical. The *P. pastoris* eukaryotic expression system is widely used to produce heterologous proteins (11–14). This technology is more effective in maintaining protein activity and function than prokaryotic systems such as *Escherichia coli* (15, 16) and it has a lower cost, higher stability and yield, and easier application than systems based on baculovirus and mammalian cells (17–20), allowing the large-scale production of recombinant proteins. Research on yeast expression systems has increased in recent years (21–24).

## Materials and methods

### Reagents and specimens

F81 cells, vesicular stomatitis virus with green fluorescent protein (VSV-GFP), and mink parvovirus virulent strain SMPV-11 were cultured in our laboratory. *E. coli* BL21 (DE3) competent cells were purchased from TransGen Biotech Co., LTD (Beijing, China). The expression vector pPICZαA and *P. pastoris* strain X33 were purchased from Invitrogen. The culture supernatant was concentrated using an ultrafiltration system with a molecular weight (MW) cutoff of 5 kDa (Vivaflow 200 Hydrosart; Sartorius, Goettingen, Germany). X33 transformants were maintained in YPD medium (1% yeast extract, 2% tryptone, 2% glucose) containing 30% (v/v) glycerol and were stored at −80°C.

Six male rats (200 g body weight) were purchased from a fur farm in Jilin Province, China. This study was approved by the Institute of Special Economic Animal and Plant Sciences of the Chinese Academy of Agricultural Sciences (CAAS; No. ISAPSAEC-2021-27M), and all sampling procedures complied with the guidelines of the Institutional Animal Care and Use (IACUS) regarding the care and use of animals for scientific purposes. This study was also approved by the Laboratory Animal Management and Welfare Ethics Committee of the Institute of Special Economic Animal and Plant Sciences.

### Vector construction

#### Synthesis of the MiIFN-α gene

Mink IFN-α cDNA is 564-bp in length. SignalP 6.0 server software analysis predicted that the 23 amino acids at the N terminal represented the signal peptide (https://services.healthtech.dtu.dk/service.php?SignalP). The encoding mature peptide gene fragment removing the signal peptide was codon-optimized and synthesized by Sangon Biotech Co. Ltd. (Shanghai, China), digested with *Eco*R I and *Xba* I, and cloned into the pUC57 vector. A 6 × His tag was added to the C-terminal region of the protein to facilitate purification.

#### Construction of the expression vector, transformation, and screening

The following primers were designed using Primer software version 5.0 to assess whether the recombinant plasmid was incorporated into the yeast genome: forward, 5′-GACTGGTTCC AATTGACAAG C-3′; reverse, 5′-GCAAATGGCA TTCTGACATC C-3′ (MiIFN-α); forward, 5′-GACTGGTTCC AATTGACAAG C-3′; reverse, 5′-GCAAATGGCA TTCTGACATC C-3′ (pPICZαA vector). These primers were synthesized by Sangon Biotech Co. Ltd.

The pUC57-MiIFN-α gene and pPICZαA were digested with *Eco*RI and *Xba*I and ligated with T4 ligase at 16°C. The ligation product was transformed into *E. coli* DH5α, and recombinant transformants were selected and cultured on low-salt LB medium containing 25 μg/mL zeocin at 37°C for 18 h. Ten colonies were plated on low-salt LB agar containing 25 μg/mL zeocin and cultured overnight at 37°C. Plasmid DNA was isolated using a TaKaRa MiniBEST Plasmid Purification kit (Kusatsu, Japan) and analyzed by PCR and 1% agarose gel electrophoresis.

Positive clones were linearized using *Sac*I and transformed into X33 by electroporation according to Invitrogen's recommendations. A pPICZαA empty vector was used as the control. Then, yeast strains containing positive clones were grown on YPD plates containing 25 μg/mL zeocin for 48–72 h. Seven colonies were selected and cultured in 2.5 mL of YPD medium containing 100 μg/mL of zeocin for 48 h. Recombinant plasmids were isolated and analyzed by PCR. PCR was performed on a 30 μL volume containing 1 μL of each primer, 15 μL of Ex Taq Premix, 13 μL of sterile water, and 1 μL of recombinant plasmids. The linearized plasmid served as a positive control.

### Expression and identification of the MiIFN-α gene

Recombinant colonies were grown to an OD_600_ of 0.6 in 100 mL of YPD medium containing 100 μg/mL of zeocin at 30°C on a rotary shaker at 200 rpm. The cells were harvested by centrifugation at 2,000 *g* for 5 min at 20–30°C, transferred to 100 mL of BMMY medium (1% yeast extract, 2% peptone, 100 mM potassium phosphate buffer pH 6.0, 1.34% yeast nitrogen base, 4 × 10^−5^% biotin, and 0.5% methanol), and grown at 30°C and 200 rpm for 17 h. Methanol (0.8%) was added every 24 h to induce protein expression, and the expression level was measured every 24 h. The optimal induction time was 12–118 h. One milliliter of the supernatant was collected at 12, 24, 36, 48, 72, 96, and 118 h and centrifuged at 10,000 rpm for 5 min. Then, 500 μL of methanol and 125 μL of chloroform were added to 500 μL of each supernatant. The test tubes were left at room temperature for 5 min and centrifuged at 12,000 rpm and 4°C for 10 min. The pellet was resuspended in 500 μL of methanol, and the suspension was centrifuged as described above. The pellet was dissolved in 50 μL of 2 × loading buffer, and proteins were separated by 12% sodium dodecyl-sulfate polyacrylamide gel electrophoresis (SDS-PAGE).

### Large-scale protein expression and purification

Yeast cells were cultured overnight in 50 mL of YPD at 30°C on a rotary shaker at 200 rpm. Subsequently, 20 mL of the culture was inoculated in 1 L of BMGY medium and grown to an OD_600_ of 2–3. Cells were harvested by centrifugation at 2,500 *g* for 15 min, transferred to 1 L of BMMY medium, and grown at 30°C for 72 h with shaking. Protein expression was induced with 0.8% methanol every 12 h. Cells were centrifuged at 2,500 *g* for 15 min, and the supernatant was collected.

The cells were cultured for 72 h and centrifuged at 12,000 g for 10 min. One liter of the supernatant was collected and filtered five times through 0.22 μm membranes and concentrated to 100 mL using an ultrafiltration system with a 5 kDa MW cutoff (Vivaflow 200 Hydrosart). The supernatant was adjusted to pH 7.4 and dialyzed using a 14 kDa MW cutoff dialysis bag overnight at 4°C in a buffer containing 20 mM sodium phosphate, 500 mM NaCl, and 20 mM imidazole pH 7.4. A nickel-nitrilotriacetic acid (Ni^2+^-NTA) affinity resin column was washed with 3 to 5 volumes of sterile deionized water and equilibrated with 5 to 10 volumes of binding buffer (20 mM sodium phosphate, 500 mM NaCl, and 30 mM imidazole). Then, proteins were loaded into the column and eluted with elution buffer (20 mM sodium phosphate, 500 mM NaCl, 500 mM imidazole, pH 7.4). Imidazole was removed by dialysis against PBS. Western blotting of the partially purified 6 × His-tagged IFN-α was performed as described previously (10). The purified protein was quantified with the bicinchoninic acid (BCA) protein assay kit using bovine serum albumin as a standard.

### Antiviral assay

The antiviral activity of MiIFN-α *in vitro* was evaluated in F81, Wish, and Vero cells using vesicular stomatitis virus (VSV) expressing green fluorescent protein and the cytopathic effect (CPE) inhibition assay (25–28). The dilution that inhibited ≥50% of the CPE was considered antiviral. Titers were converted to international units (IUs) using a reference standard. F81 cells were subcultured in modified Eagle's medium (MEM) containing 8% fetal calf serum at 37°C and 5% CO_2_ until full confluence. The medium was discarded, and cells were digested with 0.25% trypsin and suspended in MEM containing 3% fetal calf serum. Suspended cells were inoculated with VSV-GFP at a ratio of 1:10 to produce a CPE >80%. The supernatant was collected and stored at −80°C.

#### Determination of the 50% tissue culture infective dose (TCID_50_)

Suspended cells were transferred to a 96-well plate (100 μL per well) and cultured in a humidified incubator with 5% CO_2_ at 37°C in the presence of 100 μL of a 10-fold dilution of VSV-GFP and mink enteritis virus (MEV SMPV-11; GenBank: KP008112). Each dilution was assayed in octuplicate. TCID_50_ was calculated using the Reed-Muench method (29, 30). The viral culture was stored at −80°C for analysis.

#### Determination of antiviral activity in F81/GFP-VSV system

A recombinant freeze-dried canine IFN-α (2 × 10^6^ IU) (Lot No. 20200503, Tianjin Ringpu Bio-Technology Co., Ltd.; Tianjin, China) was used as a standard. F81 cells were seeded in 96-well plates and incubated in a humidified incubator with 5% CO_2_ at 37°C until full confluence. The medium was removed, and 100 μL of MiIFN-α diluted 4-fold was added to each well. Experiments were performed in quadruplicate. Different dilutions of the standard, cell control, and virus control were used. The culture medium was removed on treatment of cells with MiIFN-α for 12 h, and cells were infected with 100 μL of 100 TCID_50_. Then, 100 μL of MEM supplemented with 2% fetal calf serum was added to the wells, and plates were cultured for 24–48 h. When the CPE in virus control wells was >85%, F81 cells were stained with 0.5% crystal violet for 30 min at 20–30°C and counted under an inverted microscope (CKX53, Olympus, Tokyo, Japan).

The F81/VSV-GFP system was used to determine the antiviral activity of MiIFN-α. One hundred microliters of the purified protein diluted 4-fold was added to each well. The plates were incubated at 37°C for 12 h in 5% CO_2_, and 100 μL of the virus suspension (TCID_50_ = 8) was added to each well. The antiviral activity of purified MiIFN-α and standards was calculated when the CPE in virus control wells was >85%.

The culture medium was removed, and each well was washed three times with 200 μL PBS. Adherent F81 cells were stained with 0.5% crystal violet for 30 min. The 96-well plate was rinsed with sterile deionized water, and 100 μL of 30% acetic acid was added to each well. The absorbance was measured at 540 nm in a microplate reader. Crystal violet is an alkaline dye that binds to DNA in the cell nucleus to stain the nuclear and render viable cells blue-violet.

#### MiIFN-α inhibits MEV replication *in vitro*

According to the CPEI method (described in section Antiviral assay), F81 cells were treated with recombinant MiIFN-α for 12 h, and the MEV SMPV-11 virus was added. Cell cultures were collected at 0, 3, 6, 12, 24, 48, 72, and 96 h after inoculation. MEV genomic DNA was extracted, and the viral content was determined by quantitative real-time PCR (qPCR). A standard curve was constructed for qPCR. GraphPad Prism 8.0 software was used for statistical analysis.

### Measurement of IFN-α concentrations in rat serum

Six male rats (weighing 200 g) were used to measure serum MiIFN-α concentrations and were assigned to an intervention and a control group (subcutaneously injected with 100 μg MiIFN-α and saline, respectively; *N* = 3 in each group). Animals fasted for 24 h before treatment. Blood was collected from the tail vein at 0 h, 15 min, 30 min, and 1, 2, 4, 6, 8, 12, 24, 36, 48, 72, 96, 108, 132, and 156 h after injection, and serum was obtained by centrifugation at 4,000 rpm for 20 min at 4°C. As no commercial mink IFN-α detection kit was available, and mink and canine IFN-α share 80.9% homology at the nucleotide sequence (10), serum MiIFN-α was quantified using canine IFN alpha-1/2 (IFN-α1/2) ELISA (Abebio, China). Western blot experiments confirmed that canine IFN-α monoclonal antibody can cross-react with mink IFN-α, and thus, we used the commercial canine IFN-α ELISA kit to detect mink IFN-α in rat serum. This assay employs a two-site sandwich ELISA to quantitate MiIFN-α in rat serum. An antibody specific for CaIFN-α was pre-coated onto a microplate. Standards and samples were pipetted into the wells, and any IFN-α present was bound by the immobilized antibody. After removing any unbound substances, a biotin-conjugated antibody specific for IFN-α was added to the wells. After washing, streptavidin-conjugated horseradish peroxidase (HRP) was added to the wells. Following a wash to remove any unbound avidin-enzyme reagent, a substrate solution was added to the wells. Color developed in proportion to the amount of IFN-α bound in the initial step. The color development was stopped, and absorbance at 450 nm was measured.

### Effect of pH and temperature on antiviral activity

Mink IFN-α samples were incubated with VSV-GFP at pH 2, 4, 6, 8, 10, and 12 at 4°C for 28 h, as described above. After treatment, the sample pH was adjusted to 7.4, and the antiviral activity was determined. Untreated samples were used as controls. Assays were also performed at 56°C to assess the effect of the temperature on antiviral activity.

### Safety experiment of recombinant MiIFN-α

Fifteen double-negative mink were detected by ADV and MEV. They were divided into three groups with five animals in each group, namely, the high-dose (MiIFN-α of 3 × 10^6^ IU/kg was injected intramuscularly), low-dose (MiIFN-α of 2 × 10^5^ IU/kg was intramuscularly injected), and control groups. The animals were fasted for 12 h before the experiment, with drinking water *ad libitum*. MiIFN-α was continuously injected for 7 d, and the same volume of saline was injected in the control group. The mink were observed for any toxic reactions and findings were recorded in detail from the first day after injection. The body temperature, volume of water drank, excreta characteristics, and behavior were measured daily and observed continuously for 14 d after injection. Blood biochemical indices were continuously observed post-injection, and changes in lymphocyte, total white blood cell, and central granulocyte counts were measured. The measured data were recorded with X ± S, and SPSS 25.0 software was used for data analysis. A *t*-test analysis was performed to assess differences between the two groups. Results with a *p*-value <0.05 were considered statistically significant. After the experiment, the minks were euthanized by anesthesia. The minks were immediately dissected. The heart, liver, spleen, lung, kidney, and intestinal tissues were taken and fixed with 4% paraformaldehyde fixative. The pathological changes were observed by hematoxylin and eosin (HE) staining after paraffin embedding.

## Results

### Construction of the expression vector

**Figure 1:**
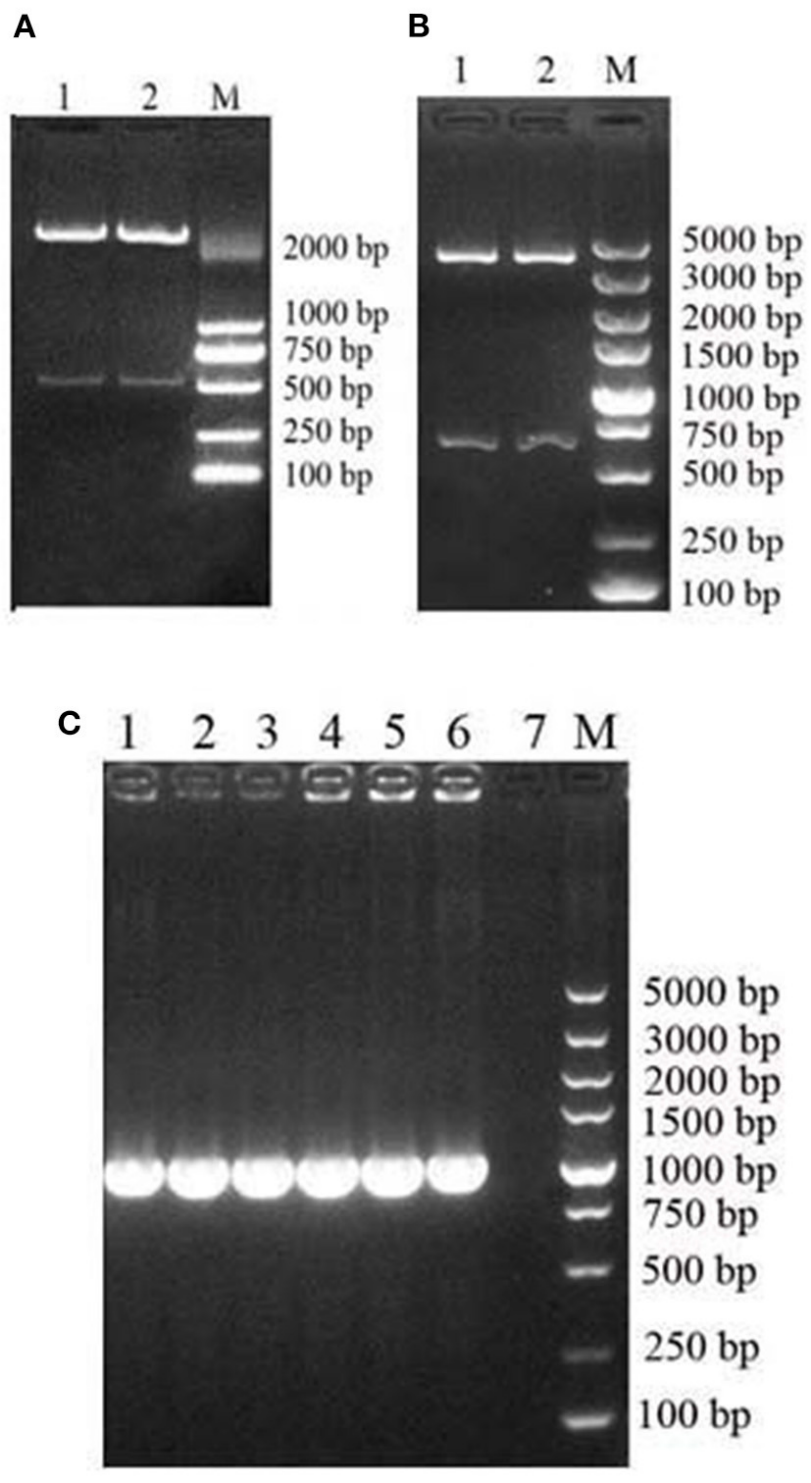
Construction and identification of an MiIFN-α recombinant yeast expression vector. **(A)** Digestion of pUC57-MiIFN-α recombinant plasmid with *Eco*R I and *Xba* I. **(B)** Identification of pPICZα-MiIFN-α recombinant plasmid by restriction enzyme digestion with *Eco*R I and *Xba*I. **(C)** PCR identification of pPICZα-MiIFN-α plasmid.

**Figure 2:**
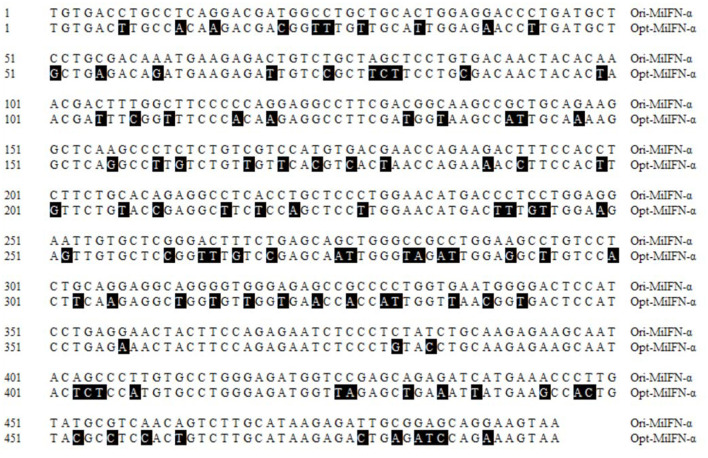
Nucleotide alignment between the original and optimized sequences. Nucleotide homology between the optimized and original sequences reached 74.8%. Black areas indicate differences in nucleotides.

The recycled fragment from the synthetic pUC57-MiIFN-α plasmid digested by *Eco*RI and *Xba*I restriction enzymes was consistent with the gene encoding mature MiIFN-α (Figure 1A). Two fragments (the pPICZα A vector 3,600 bp and synthetic gene 510 bp) were observed after *Eco*RI and *Xba*I digestion (Figure 1B). Sequencing results showed that the recombinant plasmid was successfully constructed (data not shown). A 495-bp gene fragment was amplified using specific primers, and a 1,080-bp product (containing the 495-bp vector and 495-bp target sequence) was amplified using AOXI primers, consistent with the theoretical size (Figure 1C). The results showed that the recombinant plasmid was successfully transformed and integrated into the yeast genome. pPICZα A-MiIFN-α plasmid sequencing results showed that the optimized codon sequences were consistent with the predicted results and shared 78.5% nucleotide homology with the original sequence (Figure 2).

### Protein expression and purification

**Figure 3:**
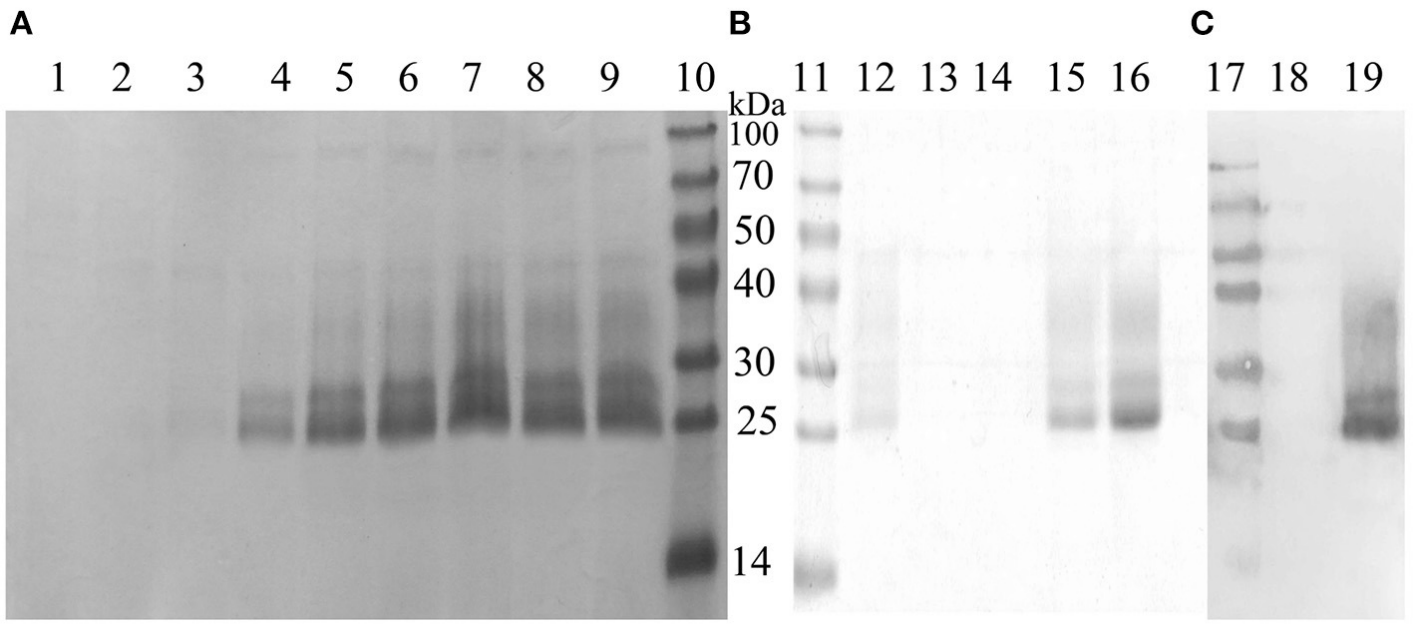
Identification of recombinant MiIFN-α expression and purification by SDS-PAGE and western blotting. **(A,B)** Yeast expression and purification of MiIFN-α was observed by SDS-PAGE. Lane 1: empty vector-induced control; Lane 2: recombinant yeast non-induced control; Lane 3: 6 h induced supernatant; Lane 4: 12 h induced supernatant; Lane 5: 24 h induced supernatant; Lane 6: 48 h induced supernatant; Lane 7: 72 h induced supernatant; Lane 8: 96 h induced supernatant; Lane 9: 108 h induction of supernatant; Lanes 10 and 11: protein molecular weight standards; Lanes 12–14: Eluted with binding buffer; Lanes 15 and 16: recombinant MiIFN-α purified by NTA-Ni chromatography at elution buffer pH 7.4. **(C)** Identification of the expressed product by western blotting. Lane 17: protein molecular weight standards; Lane 18: empty vector induced control; Lane 19: purified target.

**Figure 4:**
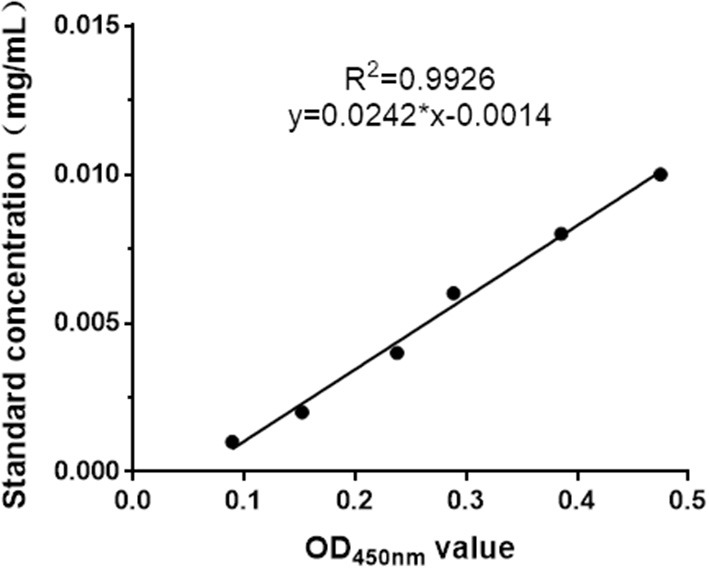
BCA standard curve drawing. BSA dilution concentrations ranged from 0.001 to 0.01 mg/mL. The BCA standard curve indicates that the linear correlation of the protein concentration and OD_450nm_ value was y = 0.0242x-0.0014 (*R*^2^ = 0.9926).

The recombinant plasmid, pPICZα A-MiIFN-α, was linearized by digestion with *Sac* I and transformed into competent X33 *P. pastoris* cells resistant to zeocin. Zeocin-resistant colonies were selected, and mink IFN-α expression was induced in recombinant transformants using 0.2, 0.4, 0.6, 0.8, 1, 1.2, and 1.6% methanol. After methanol induction, 12% SDS-PAGE analysis showed that MiIFN-α was expressed in the yeast culture supernatant. Protein levels were highest at 72 h post-induction with 0.8% methanol. The induced protein accounted for more than 80% of the total protein in the culture supernatant. The MW of MiIFN-α was ~25 kDa (5 kDa larger than expected) (Figure 3). The concentration of purified MiIFN-α was 0.095 mg/mL, according to the BCA standard curve (Figure 4); the yield of mink IFN-α protein reached 2.85 mg/mL in the culture supernatant.

### Antiviral activity of MiIFN-α

**Figure 5:**
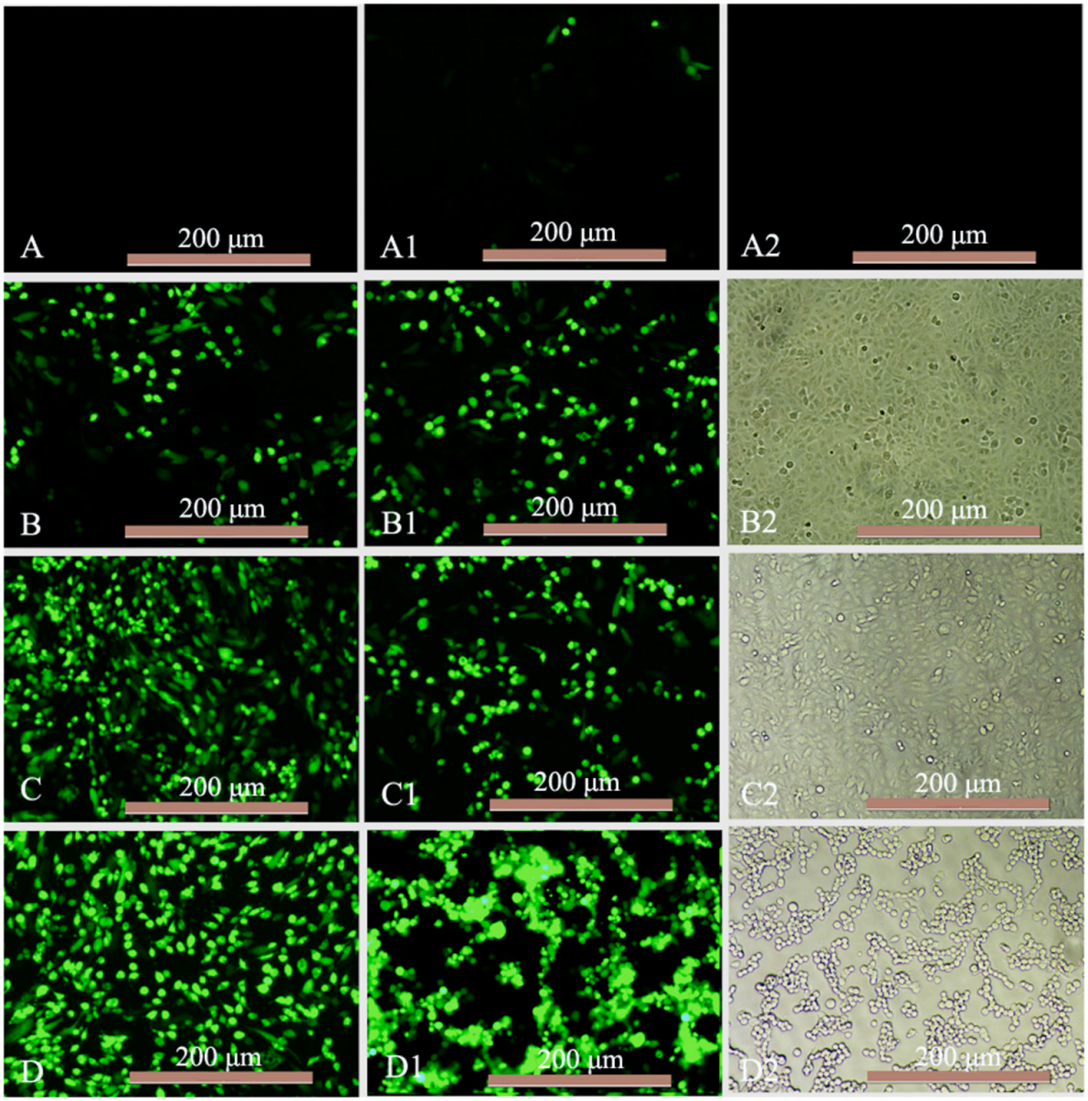
Antiviral activity of MiIFN-α against VSV-GFP in F81 cells. **(A–D)** Changes in the green fluorescence intensity indicating the antiviral activity of the canine IFN-α standards of 488.28 IU/mL (4^6^-fold), 122.07 IU/mL (4^7^-fold), 30.52 IU/mL (4^7^-fold), and 7.63 IU/mL (4^7^-fold). **(A1)** MiIFN-α 0.36 ng/mL(4^6^-fold). **(B1)** MiIFN-α 0.091 ng/mL(4^7^-fold). **(C1,C2)** MiIFN-α 0.023 ng/mL (47-fold). **(A2,B2)** Uninfected cells. **(D1,D2)** Virus controls.

The antiviral activity of mink IFN-α expressed in X33 *P. pastoris* was evaluated using the CPEI method in F81/VSV-GFP and F81/MEV systems. F81 cells were stimulated with the purified IFN-α for 12 h, and multiple antiviral proteins were induced to inhibit virus replication. VSV-GFP TCID^50^ (=8) was calculated using the Reed-Muench method. F81 cells were observed after treatment with serial 4-fold dilutions of standard or MiIFN-α and infection with 100 μL VSV-GFP (TCID_50_ = 8). CPE in F81 cells was more than 85% in virus-infected cells, when the virus was inoculated for 40 h. Infected cells were round and gathered in clusters at the bottom of the plate (Figure 5D2); the green fluorescence was strong in these cells (Figure 5D1). No CPE and green fluorescence was observed in control cells (Figures 5A2,B2). Standard concentration ≥ 488.28 IU/mL (Figure 5A) and MiIFN-α concentration ≥ 0.36 ng/mL (Figure 5A1) significantly inhibited the CPE. Standard concentration ≤ 122.07 IU/mL (Figures 5B–D) and MiIFN-α concentration ≤ 0.091 ng/mL (Figures 5B1,C1, C2) did not inhibit the CPE, and the fluorescence intensity increased with the increase in the MiIFN-α dilution ratio. An MiIFN-α concentration of 6.4 × 10^7^ IU/mL effectively inhibited VSV-GFP; the effective concentration was ~6.7 × 10^8^ IU/mg. The results of CPE and fluorescence assays were concordant.

**Figure 6:**
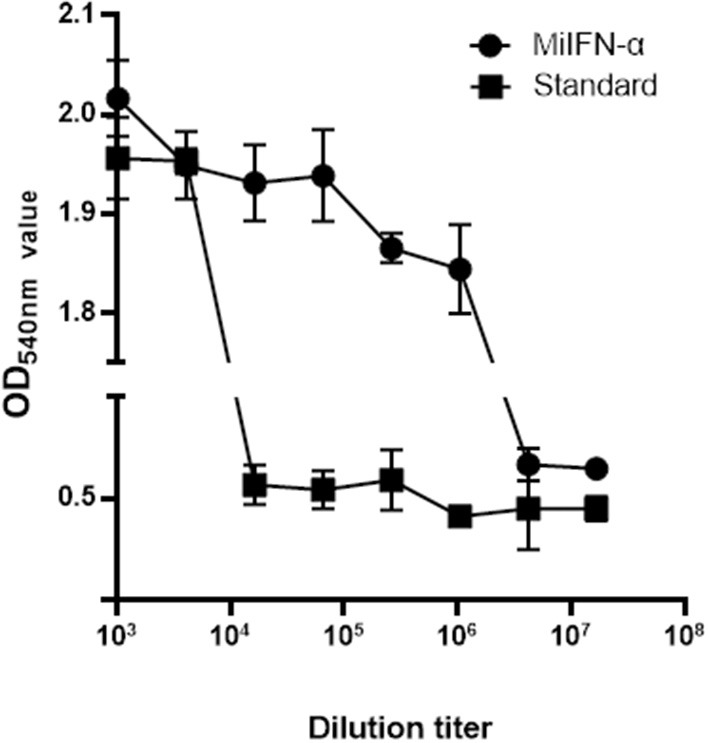
Observation of cell staining with crystal violet. Adherent F81 cells were stained with 0.5% crystal violet, which rendered the viable cells blue-violet; the non-colored dead cells were washed away with the effluent. Results of absorbance detection at 540 nm show that the antiviral activity of standard canine IFN-α (2 × 10^6^ IU/mL) decreased significantly at 4^6^ dilutions and that of mink IFN-α slowly decreased at 4^9^ dilutions.

Cells were stained with 0.5% crystal violet and attached to the 96-well plate; dead cells were washed away. The results of antiviral activity detection were consistent with those of the fluorescence observation (Figure 6).

### Measurement of serum IFN-α concentration

**Figure 7:**
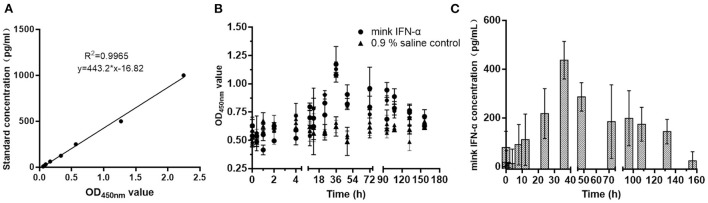
Detection of the metabolism of MiIFN-α in rats. **(A)** Standard curve drawing of canine IFN-α ELISA kit. **(B)** OD_450nm_ absorbance at different time points. **(C)** Residual concentration of interferon-α at different time points in rats.

A single 100-μg dose of MiIFN-α was injected subcutaneously into rats. Serum MiIFN-α levels were quantified using the canine IFN-α ELISA standard curve (Figure 7A). Mink IFN-α concentration in rat serum was significantly increased compared with that in the 0.9% saline control at 8 h post-injection and peaked at 36 h post-injection (Figures 7B,C), reaching 482.52 pg/mL. Subsequently, the concentration decreased slowly and returned to a normal level at 158 h post-injection.

### MiIFN-α inhibits MEV replication *in vitro*

**Figure 8:**
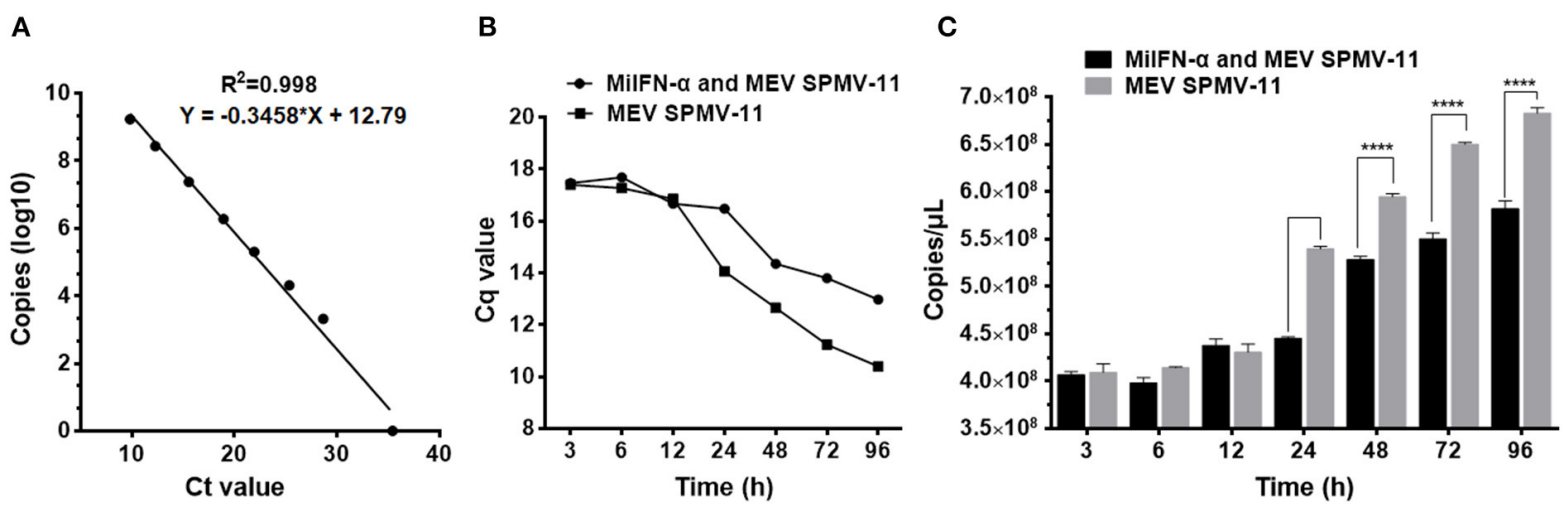
MiIFN-α inhibits MEV SMPV-11 replication *in vitro*. **(A)** MEV Standard curve of quantitative real-time PCR. **(B)** Changes in Cq values. **(C)** Changes in copy number. ****, *P* ≤ 0.0001.

Establishment of qPCR standard curve using pMD18-T/VP2 as a standard plasmid showed that the linear correlation of *Ct* value and copies (log10) was y = −0.3458x + 12.79 (*R*^2^ = 0.998) (Figure 8A). F81 cells were incubated with 100 μL of 0.95 mg/mL MiIFN-α for 12 h and incubated with MEV SMPV-11. Cell cultures were collected at 0, 3, 6, 12, 24, 48, 72, and 96 h after IFN-α-treated cells were challenged with MEV. The copy number of the MEV SMPV-11 gene was determined using a standard curve. There were no significant differences between the MiIFN-α treatment and untreated groups from 3 to 12 h after F81 cells were challenged with the MEV SMPV-11 strain (*p* > 0.05) (Figure 8B). However, the number of viral copies decreased significantly at 24, 48, 72, and 96 h in the MiIFN-α treatment group compared to that in the MEV SMPV-11 control group (*p* < 0.0001) (Figure 8C); in other words, MiIFN-α significantly inhibited MEV replication in F81 cells.

### Antiviral activity of MiIFN-α under different pH conditions

The antiviral activity of MiIFN-α was significantly affected by pH under our experimental conditions, except at pH 6 and pH 8. The antiviral activity in the pH 2, pH 4, pH 10, and pH 12 treatment groups reached 1.97 × 10^5^ IU/mL, 2.62 × 10^5^ IU/mL, 2.58 × 10^5^ IU/mL, and 1.98 × 10^5^ IU/mL, respectively.

**Figure 9:**
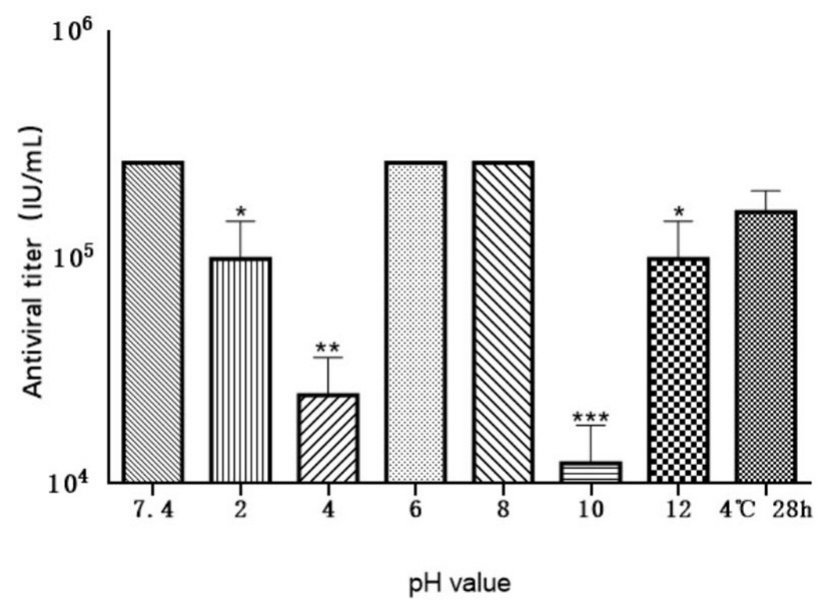
Effect of various pH levels on the antiviral activity of MiIFN-α. Antiviral activity of mink IFN-α in a F81/VSV system treated at 4°C for 24 h at pH 2, 4, 6, 7.4, 8, 10, 12, and for 28 h at pH 7.4; *p* < 0.01. *, *P* ≤ 0.05; **, *P* ≤ 0.01; ***, *P* ≤ 0.001.

A significant difference in antiviral activity was observed between the pH 2 and pH 12 treatment groups and the untreated group (*p* < 0.01, *p* < 0.01). The activities in the pH 4 and pH 10 treatment groups reached 4.9 × 10^4^ IU/mL and 2.5 × 10^4^ IU/mL, respectively, with extremely significant differences (0.001 < *p* < 0.01, 0.001 < *p* < 0.01) compared to the pH 7.4 treatment group. No significant difference was noted at pH 6, pH 8, and pH 7.4 after storage at 4°C for 28 h, confirming the chemical instability of the expressed protein (Figure 9).

#### Confirmation of mink IFN-α safety

MiIFN-α 1 × 107 IU/kg was intramuscularly injected into mink. Body temperature of mink in the injected group did not change significantly compared with that in the mock control group, fluctuating within 38.8–39.5°C (*p* > 0.05). Post-administration, changes in lymphocytes, white blood cells, and central granulocytes in the fingertips of the hind limbs of mink were monitored; compared with the control group, all groups showed no significant difference (*p* > 0.05). High-dose recombinant mink IFN-α was injected continuously for 7 d, and the main organs of mink were collected on the 14th day. After HE staining, no obvious pathological changes were observed in the tissues including heart, liver, spleen, lung, and kidney, indicating that recombinant mink IFN-α is safe for use in mink.

## Discussion

IFNs are glycoproteins with antiviral, antitumor, and immunomodulatory effects (31) and are known to reduce the replication of the influenza virus in the chick embryo chorioallantoic membrane (32). Type I IFNs have higher antiviral activity than other IFN types, and the former has been used to treat viral diseases. Several IFNs have been characterized to date (26, 33, 34). IFN has been expressed successfully in *E. coli*, and its antiviral activity has been measured; nonetheless, protein accumulation in inclusion bodies has limited its purification (10).

Mink IFN-α proteins have been successfully expressed in *E. coli* expression systems, and most of these proteins have two glycosylation sites. We attempted to induce the soluble expression of MiIFN-α in the prokaryotic expression vectors pPROEX HTa, pET28a, and pCold II at 16–28°C. However, this protein was expressed in inclusion bodies, limiting purification. In addition, the complexity of the baculovirus expression system hinders the large-scale production of IFNs. Therefore, the *P. pastoris* expression system was used in the present study to produce MiIFN-α, and this protein was secreted in a soluble form. The methylotrophic yeast *P. pastoris* produces large amounts of intra- and extracellular recombinant proteins in their soluble forms. Endogenous signal peptides mediate the secretion of recombinant proteins in *P. pastoris*. The pPICZα vector secretes exogenous proteins into the extracellular environment, facilitating purification (35, 36). Some soluble proteins are N-glycosylated during expression, and the degree of glycosylation is similar to that of native proteins (24, 25). The advantages of this system include its high yield, proper protein folding and post-translational modifications, simple operation, low cost, and suitability for large-scale production (37–40).

The MW of the target protein, which was 5 kDa higher than expected, may be due to glycosylation or incomplete cleavage of the α signal peptide. Previously, it was reported that the MW of protein expressed by yeast was higher than the predicted MW, possibly owing to incomplete cleavage of the α signal peptide sequence during secretion or glycosylation of the expressed protein (13, 41–43). However, MiIFN-α had high antiviral activity, and its level peaked in rat serum at 36 h after injection. In F81 cells, the replication of the virulent MEV strain SMPV-11 was significantly inhibited, providing a basis for research in to the *in vivo* applications of antiviral treatment of mink. A new study from our team is underway to assess whether PEGylation and nano-encapsulation prolong the half-life of MiIFN-α in rats.

The protein yield in shake flasks is lower than that in fermentation tanks because of the small volume and limited oxygen supply in the former. Therefore, the supernatant was concentrated by more than 10-fold in our samples, and the purity of MiIFN-α was 95%. These findings pave the way for the large-scale production and clinical application of this cytokine.

## Data availability statement

The datasets presented in this study can be found in online repositories. The names of the repository/repositories and accession number(s) can be found in the article/supplementary material.

## Ethics statement

The animal experiments in this study were approved by Specialties, Chinese Academy of Agricultural Sciences (Beijing, China). This study was also approved (No. ISAPSAEC-2021-27M) by the Laboratory Animal Management and Welfare Ethics Committee of the Institute of Special Economic Animal and Plant Sciences and the Chinese Academy of Agricultural Sciences, and all sampling procedures complied with the Institutional Animal Care and Use (IACUS) guidelines regarding the care and use of animals for scientific purposes. Written informed consent was obtained from the owners for the participation of their animals in this study.

## Author contributions

HZ and SLu conceived and designed the experiments, analyzed the data, and wrote the manuscript. DZh and HL conducted the experiments. DZo, BH, and SLi carried out the statistical analyses. SLu provided guidance and revised the manuscript. All authors have read and approved the final manuscript.

## Funding

This study was supported by the National Natural Science Foundation of China (NSFC, No. 32072943) and the Science, Technology Innovation Project of Chinese Academy of Agricultural Sciences (No. CAAS-ASTIP-2016-ISASPS), the Science and Technology Development Program of Jilin Province (20190401006NY), and the Fundamental Research Funds for the Central Universities (45121031B007).

## Conflict of interest

The authors declare that the research was conducted in the absence of any commercial or financial relationships that could be construed as a potential conflict of interest.

## Publisher's note

All claims expressed in this article are solely those of the authors and do not necessarily represent those of their affiliated organizations, or those of the publisher, the editors and the reviewers. Any product that may be evaluated in this article, or claim that may be made by its manufacturer, is not guaranteed or endorsed by the publisher.
